# Systemic immune-inflammatory index and its association with female sexual dysfunction, specifically low sexual frequency, in depressive patients: Results from NHANES 2005 to 2016

**DOI:** 10.1097/MD.0000000000038151

**Published:** 2024-05-31

**Authors:** Guangwei Qing, Hao He, Minghao Lai, Xue Li, Yan Chen, Bo Wei

**Affiliations:** a Department of Psychiatry, Jiangxi Mental Hospital & Affiliated Mental Hospital of Nanchang University, Nanchang, Jiangxi, China; b Third Clinical Medical College, Nanchang University, Nanchang, Jiangxi, China; c Nanchang City Key Laboratory of Biological Psychiatry, Jiangxi Provincial Clinical Research Center on Mental Disorders, Jiangxi Mental Hospital, Nanchang, Jiangxi, China.

**Keywords:** female sexual dysfunction, sexual frequency, sexual health, systemic immune-inflammatory index

## Abstract

Sexual dysfunction, particularly in females, is a complex issue influenced by various factors, including depression and inflammation. The Systemic immune-inflammation index (SII), an inflammatory biomarker, has shown associations with different health conditions, but its relationship with female sexual dysfunction (FSD) remains unclear. This study aimed to investigate the association between SII and FSD in the context of depression, utilizing low sexual frequency as an assessment indicator. Data from the National Health and Nutrition Examination Survey (NHANES) 2005 to 2016, involving 1042 depressed female participants, were analyzed. FSD, indicated by low sexual frequency, and SII, derived from complete blood count results, were assessed. Logistic regression and subgroup analyses were conducted, considering demographic and health-related factors. A total of 1042 individuals were included in our analysis; 11.5163% of participants were categorized as having FSD, which decreased with the higher SII tertiles (tertile 1, 13.8329%; tertile 2, 13.5447%; tertile 3, 7.1839%; p for trend < 0.0001). Multivariate linear regression analysis showed a significant negative association between SII and FSD [0.9993 (0.9987, 0.9999)]. This negative association in a subgroup analysis is distinctly and significantly present in the Mexican American subgroup [0.9959 (0.9923, 0.9996)], while it does not reach statistical significance in other racial categories. Furthermore, the association between SII and FSD was nonlinear; using a 2-segment linear regression model, we found a U-shaped relationship between SII and FSD with an inflection point of 2100 (1000 cells/µL). In summary, in depressed individuals, a higher SII is independently associated with a decreased likelihood of FSD, emphasizing the potential role of inflammation in female sexual health.

## 1. Introduction

Within the framework of the biopsychosocial model, female sexual dysfunctions (FSD) manifest in women across diverse age groups and stem from a myriad array of causative factors.^[1]^ The classification system for these dysfunctions has evolved from a linear categorization, which includes sexual desire, arousal, orgasm, and pain disorders, into a more intricate and interconnected framework.^[2]^ The prevalence of FSD, as indicated by information extracted from the National Health and Social Life Survey (NHSLS) dataset, is estimated at 43%, notably exceeding that of men, with 43% of women reporting sexual problems, in contrast to 31% of men.^[3]^ Disruptions in sexual desire and alterations in the psychophysiological processes integral to the sexual response cycle may result in significant distress and interpersonal challenges.^[4]^ Psychiatric disorders represent a pivotal risk factor in the context of FSD.^[5]^ The existing literature indicates an association between depression, anxiety, and FSD, underscoring the intricate relationship between sexual health and mental well-being, potentially surpassing the significance of factors such as physical function, stress, or chronological age.^[6,7]^

Depressive disorder, characterized by a wide spectrum of presentations and a diverse constellation of associated symptoms, is increasingly acknowledged as a significant mental health challenge.^[8,9]^ Worldwide, approximately 4.4% of the population is estimated to suffer from major depressive disorder. The World Health Organization (WHO) has recognized it as the foremost contributor to worldwide disability and non-lethal health-related detriments.^[10]^ Numerous prospective cohort investigations have explored the interrelation between sexual dysfunction and susceptibility to depression, along with the reciprocal association linking depression and vulnerability to sexual dysfunction.^[11,12]^ Hence, the management of sexual behavior in individuals with depression holds profound clinical importance.

The Systemic immune-inflammation index (SII) stands as a dependable and innovative inflammatory biomarker, formulated through the multiplication of platelet count by neutrophil count, subsequently divided by the lymphocyte count.^[13]^ The SII index, originally employed for prognostic assessments in solid cancer patients and cardiovascular disease, is now recognized as a reliable indicator of inflammatory status.^[14,15]^ Based on prior research, heightened inflammation emerges as a significant risk factor for sexual dysfunction. For instance, Tierney et al observed that the presence of inflammation could potentially disrupt female sexual desire and arousal through a combination of direct neural pathways and indirect influences stemming from endocrine, endothelial, and social/behavioral factors.^[16]^ Fontes-Cal et al have reported the correlations between inflammation, cardiovascular disease (CVD), and sexual arousal dysfunction within the cohort of women.^[17]^ Nevertheless, the association between the inflammatory biomarker SII and FSD remains inconclusive.

Therefore, our investigation sought to examine the correlation between SII and FSD in individuals with depression, utilizing low sexual frequency as an indicator, using data from the US National Health and Nutrition Examination Survey (NHANES). We hypothesized that a higher SII index would be associated with a decreased likelihood of FSD in individuals with depression.

## 2. Methods

### 2.1. Study population

Cross-sectional data were obtained from the NHANES, a research initiative conducted by the National Center for Health Statistics (NCHS) aimed at evaluating the health and nutritional status of the United States populace. The investigation employed an advanced multistage probability design to establish a nationally representative cohort comprising non-institutionalized residents in the United States.^[18]^ All study protocols within the NHANES framework obtained approval from the Research Ethics Review Board of the NCHS, and written informed consent was acquired from all participants surveyed. Detailed information can be accessed publicly on the CDC website at https://www.cdc.gov/nchs/nhanes/.

**Figure 1.**
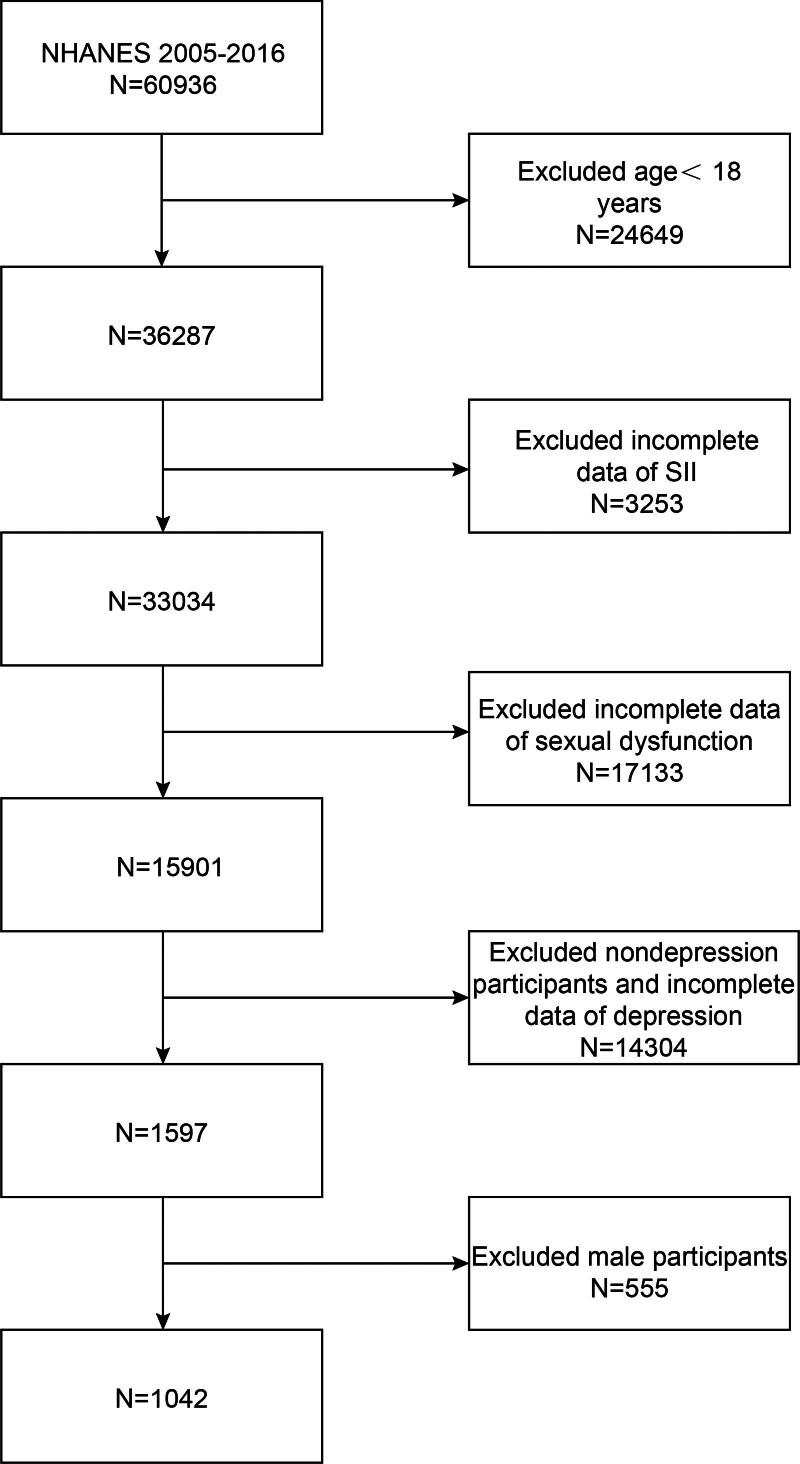
Flowchart of participant selection. NHANES = National Health and Nutrition Examination Survey, SII =systemic immune-inflammation index.

The study population consisted of participants from the NHANES 2005 to 2016 dataset, and our analysis encompassed individuals with complete datasets for FSD and SII. In the initial phase, a comprehensive cohort comprising 60,936 participants was recruited for the study. After exclusions for individuals aged <18 years (n = 24,649), missing SII data (n = 3253), incomplete sexual dysfunction data (n = 17,133), non-depressive participants, and incomplete depression data (n = 14,304), as well as male participants (n = 555), a final analysis cohort of 1042 eligible participants was established (Fig. 1).

### 2.2. Assessment of sexual dysfunction

In this study, FSD served as the designated outcome variable, with sexual frequency utilized as an established indicator for its assessment, as demonstrated in prior research.^[19]^ The examination of sexual behavior predominantly revolves around analyzing sexual interaction frequency.^[20]^ This frequency of sexual encounters, while pivotal, albeit imperfect, stands as a fundamental parameter consistently included in scales designed to assess FSD.^[21]^ FSD was defined as having 11 or fewer sexual incidents in the past 12 months, a cutoff for clinically relevant FSD based on previous research.^[19]^

### 2.3. Definition of SII

The SII was the primary exposure variable in our analysis, calculated based on the results of the complete blood count test. Specifically, it was determined as platelet count multiplied by neutrophil count and then divided by lymphocyte count, as established in previous studies.^[22]^ Lymphocyte, neutrophil, and platelet counts were evaluated utilizing an automated hematology analyzer (Coulter DxH 800 analyzer) and reported in units of 10³ cells/mL.^[23]^

### 2.4. Assessment of depression individuals

The assessment of depression involved using the patient health questionnaire (PHQ-9), a 9-item screening instrument designed to inquire about the frequency of depressive symptoms experienced over the preceding 2-week period.^[24]^ Each query is graded on a scale from “0” (absent) to “3” (almost daily), resulting in total questionnaire scores within the range of 0 to 27. A threshold of 10 or higher was employed to indicate the presence of depressive symptoms.^[25]^

The baseline variables within this investigation encompassed a range of demographic and health-related factors. These included age (years), race (Mexican American/other Hispanic/non-Hispanic White/non-Hispanic Black/other races), marital status (married or living with partner/Widowed, divorced, separated, and never married), educational level (<9th grade/9–11th grade/high school grad/GED or equivalent/some college or AA degree/college graduate or above), body mass index (BMI) (kg/m^2^), waist circumference (cm), income-to-poverty ratio (%), smoking status, diabetes, hypertension, pregnancy, number of pregnancy and deliveries, age of first sexual intercourse (year), menstruation, hysterectomy, birth control pills, female hormones, and lower sexual frequency (≤11 encounters/years). Detailed information on the measurement protocols for these study variables is publicly available on the official website of the National Center for Health Statistics (www.cdc.gov/nchs/nhanes/).

### 2.5. Statistical analysis

All statistical analyses were conducted following the guidelines set by the Centers for Disease Control and Prevention, incorporating the utilization of pertinent NHANES sampling weights to account for the intricacies inherent in multistage cluster survey designs.^[26]^

Continuous variables were concisely summarized by presenting their means accompanied by the standard error (SE), while categorical parameters were depicted using their respective proportions. For continuous parameters, either a weighted Student *t*-test or, for categorical variables, a weighted chi-square test was employed to assess distinctions among the participant groups stratified based on SII tertiles.

Multivariate logistic regression analyses were performed to investigate the autonomous association between SII and FSD across 3 distinct models. Model 1 involved no covariate adjustments, while Model 2 incorporated adjustments for age and race. Model 3 extended the adjustments to include age, race, marital status, educational attainment, BMI, income-to-poverty ratio, smoking status, diabetes, and menstrual status. To investigate the potential nonlinear association between SII and FSD, a penalized spline method for smooth curve fitting was employed, and weighted Generalized Additive Model (GAM) regression was conducted. Subsequently, subgroup analyses were carried out stratified by various factors, including age, race, BMI, marital status, hypertension, diabetes, and menstruation, using stratified multivariate regression analysis. An interaction term was introduced to assess heterogeneity among these subgroups, and its significance was tested via the log-likelihood ratio test model. Additionally, a model for analyzing threshold effects was utilized to elucidate the association and identify potential inflection points in the relationship between SII and FSD.

The threshold for statistical significance in all analyses was set at a level of *P* < .05. All statistical procedures were conducted using Empower software (www.empowerstats.com; X&Y Solutions, Inc., Boston, MA) and R version 3.4.3 (http://www.R-project.org, The R Foundation).

## 3. Results

### 3.1. Baseline characteristics of participants

The study encompassed a cohort of 1042 participants, all of whom were female and presented symptoms of depression, with an average age of 37.9203 ± 11.4345 years. Among the participants, 11.51% were categorized as having FSD, and this prevalence decreased with higher SII tertiles. Specifically, the prevalence of FSD was 13.8329% in tertile 1, 13.5447% in tertile 2, and 7.1839% in tertile 3, respectively. Significant intergroup variations were evident across the 3 SII tertiles concerning race, BMI, waist circumference, pregnancy history, delivery count, menstruation, and reduced sexual frequency (≤11 encounters per year) (all *P* < .05). Participants with higher SII levels were non-Hispanic White, had a BMI ≥ 30, larger waist circumference, had been pregnant, experienced abnormal menstruation, and reported higher sexual frequency in our study (all *P* < .05). Table 1 presents the weighted baseline demographic characteristics of the study participants.

**Table 1:** Characteristics of the study population.

Systemic immune-inflammation index	Tertile 1 (N = 347)	Tertile 2 (N = 347)	Tertile 3 (N = 348)	*P* value
Age(yr)	37.7291 ± 12.6658	37.3429 ± 11.2404	38.6868 ± 10.2635	.258
Race, % (SE)				<.001
Mexican American	49 (14.1210)	60 (17.2911)	55 (15.8046)	
Other Hispanic	50 (14.4092)	47 (13.5447)	38 (10.9195)	
Non-Hispanic White	116 (33.4294)	144 (41.4986)	167 (47.9885)	
Non-Hispanic Black	112 (32.2767)	70 (20.1729)	61 (17.5287)	
Other race	20 (5.7637)	26 (7.4928)	27 (7.7586)	
Marital status, % (SE)				.138
Married or living with partner	164 (49.8480)	182 (53.8462)	195 (57.5221)	
Widowed, divorced, separated, and never married	165 (50.1520)	156 (46.1538)	144 (42.4779)	
Education level, % (SE)				.110
<9th grade	28 (8.5106)	31 (9.1716)	16 (4.7198)	
9–11th grade	69 (20.9726)	79 (23.3728)	77 (22.7139)	
High School Grad/GED or Equivalent	74 (22.4924)	77 (22.7811)	92 (27.1386)	
Some college or AA degree	129 (39.2097)	107 (31.6568)	118 (34.8083)	
College graduate or above	29 (8.8146)	44 (13.0178)	36 (10.6195)	
Body mass index (kg/m^2^), % (SE)				.037
<25	100 (28.9855)	103 (30.2053)	80 (23.2558)	
25 to <30	79 (22.8986)	82 (24.0469)	67 (19.4767)	
≥30	166 (48.1159)	156 (45.7478)	197 (57.2674)	
Waist circumference (cm)	97.3908 ± 17.8687	97.7102 ± 17.3357	104.3439 ± 20.2879	<.001
Income-to-poverty ratio (%)	1.6393 ± 1.3261	1.7200 ± 1.4318	1.8281 ± 1.4949	.473
Smoking status, % (SE)				.136
Yes	178 (52.6627)	194 (56.8915)	206 (60.2339)	
No	160 (47.3373)	147 (43.1085)	136 (39.7661)	
Diabetes, % (SE)				.198
Yes	32 (9.2219)	33 (9.5376)	44 (12.7168)	
No	311 (89.6254)	309 (89.3064)	293 (84.6821)	
Borderline	4 (1.1527)	4 (1.1561)	9 (2.6012)	
Hypertension, % (SE)				.087
Yes	111 (31.9885)	101 (29.1066)	128 (36.8876)	
No	236 (68.0115)	246 (70.8934)	219 (63.1124)	
Ever been pregnant, % (SE)				.024
Yes	277 (84.9693)	295 (87.2781)	310 (91.7160)	
No	49 (15.0307)	43 (12.7219)	28 (8.2840%)	
Number of pregnancy	3.6583 ± 2.6345	3.5085 ± 1.8606	3.4662 ± 2.1247	.599
Number of deliveries	1.9604 ± 1.6700	1.9966 ± 1.5152	1.7068 ± 1.4637	.044
Age of first sexual intercourse(yr)	16.4380 ± 3.5210	16.4957 ± 4.7894	16.0172 ± 2.8089	.823
Menstruation, % (SE)				.002
Abnormal menstruation	221 (64.0580)	254 (73.1988)	264 (75.8621)	
Regular menstruation	124 (35.9420)	93 (26.8012)	84 (24.1379)	
Hysterectomy, % (SE)				.738
Yes	58 (18.4127)	51 (16.0883)	54 (17.5325)	
No	257 (81.5873)	266 (83.9117)	254 (82.4675)	
Ever taken birth control pills, % (SE)				.374
Yes	252 (72.8324)	268 (77.2334)	265 (76.1494)	
No	94 (27.1676)	79 (22.7666)	83 (23.8506)	
Female hormones, % (SE)				.999
Yes	42 (12.8049)	43 (12.7596)	43 (12.6844)	
No	286 (87.1951)	294 (87.2404)	296 (87.3156)	
Lower sexual frequency (≤11 encounters/yr), % (SE)				.008
Yes	48 (13.8329)	47 (13.5447)	25 (7.1839)	
No	299 (86.1671)	300 (86.4553)	323 (92.8161)	

AA = associate of arts, BMI = body mass index, GED = general educational development.

### 3.2. Higher SII associated with a lower likelihood of FSD

Table 2 displays the outcomes of the multivariable regression analysis. A negative correlation between SII and FSD was observed in our study. In the unadjusted model [0.9993 (0.9987, 0.9999)], a substantial negative association was observed between the SII and FSD, indicating that each unit increase in the SII score was associated with a 0.07% decreased risk of increased FSD. However, when age and race variables were introduced as adjustments in Model 2 [0.9994 (0.9988, 1.0000)], the initially significant negative correlation no longer remained statistically significant. Furthermore, in Model 3, when all covariates were taken into account, the association between SII and FSD lost statistical significance [0.9994 (0.9988, 1.0001)]. In contrast to participants in the lowest SII tertile, those in the highest SII tertile exhibited a significantly reduced risk of FSD, reaching statistical significance (OR = 0.5146; 95% CI, 0.2839, 0.9329, *P* = .0286) (Table 3).

**Table 2:** The threshold effect of the systemic immune-inflammatory index on female sexual dysfunction in depressed patients stratified by smoking status was analyzed using a 2-part linear regression model.

	Smoking status	
	Yes	No
Fitting by standard linear model		
OR (95% CI)	0.9990 (0.9981, 1.0000)	0.9997 (0.9987, 1.0007)
*P* value	.0451	.5529
Fitting by 2-piecewise linear model		
Breakpoint (K)	2100	2100
OR1(<K)	0.9984 (0.9972, 0.9995) 0.0058	0.9999 (0.9987, 1.0012) 0.9009
OR2(>K)	1.0066 (1.0000, 1.0132) 0.0489	0.7712 (0.0000, inf.) 0.987
Logarithmic likelihood ratio test *P* value	.040	.450

Age, race, marital status, education level, body mass index, income-to-poverty ratio, diabetes, and menstruation were adjusted.

95% CI = 95% confidence interval, OR = odds ratio.

**Table 3:** The association between female sexual dysfunction and systemic immune-inflammatory index in individuals with depression.

	Crude model (Model 1)	Partially adjusted model (Model 2)	Fully adjusted model (Model 3)
	OR (95% CI) *P* value	OR (95% CI) *P* value	OR (95% CI) *P* value
Systemic immune-inflammatory index	0.9993 (0.9987, 0.9999) .025118	0.9994 (0.9988, 1.0000) .062930	0.9994 (0.9988, 1.0001) .098166
Systemic immune-inflammatory index tertiles			
Tertile 1	Reference	Reference	Reference
Tertile 2	0.9759 (0.6330, 1.5046) .912066	1.1049 (0.7041, 1.7336) .664392	1.0887 (0.6580, 1.8013) .740785
Tertile 3	0.4821 (0.2900, 0.8016) .004913	0.5255 (0.3112, 0.8874) .016091	0.5146 (0.2839, 0.9329) .028600
P for trend	0.9988 (0.9979, 0.9996) .003703	0.9989 (0.9981, 0.9998) .011144	0.9989 (0.9979, 0.9998) .022194

Model 1, no covariates were adjusted. Model 2, age and race were adjusted. Model 3, age, race, marital status, education level, BMI, income-to-poverty ratio, smoking status, diabetes, and menstruation were adjusted.

95% CI = 95% confidence interval, BMI = body mass index, OR = odds ratio.

### 3.3. Nonlinear relationship between SII and FSD

**Figure 2.**
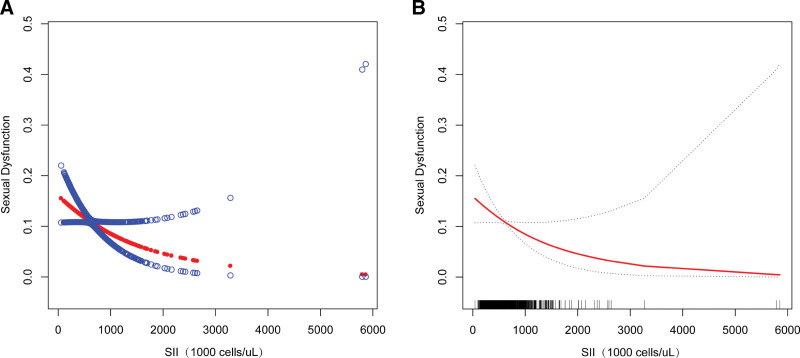
The association between SII and FSD. (A) The solid red line represents the smooth curve fit between variables. (B) Blue bands represent the 95% confidence interval from the fit. SII = systemic immune-inflammation index.

**Figure 3.**
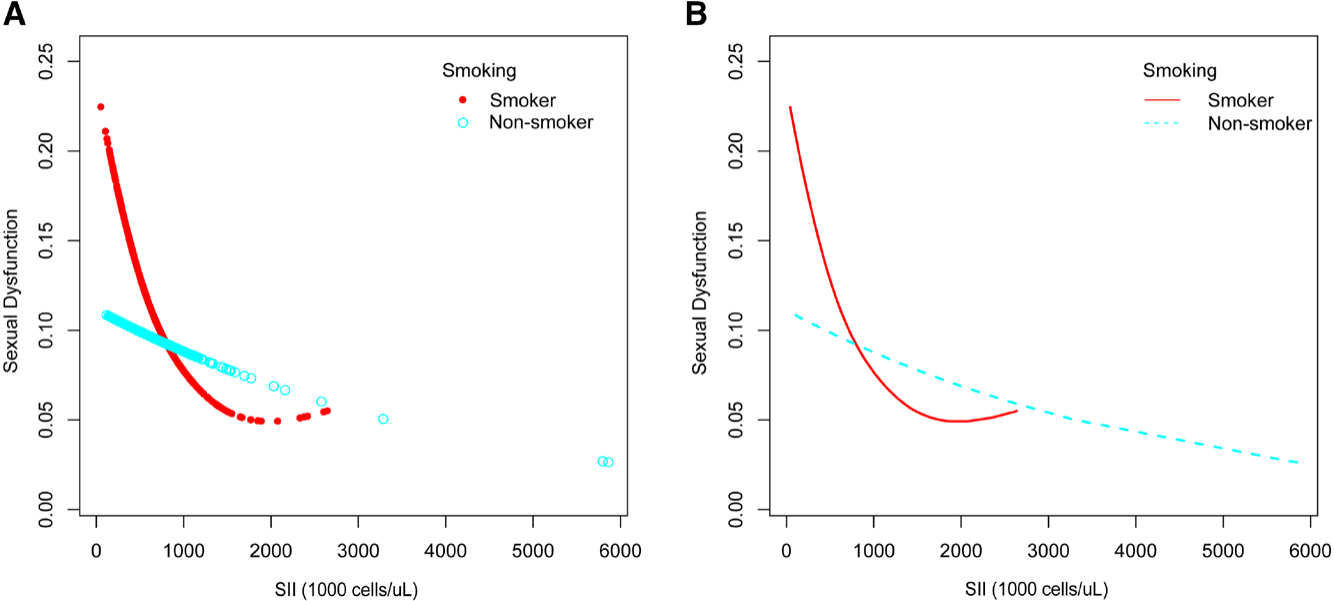
The association between SII and FSD is stratified by smoking status. SII = systemic immune-inflammation index.

Smooth curve fits and weighted generalized additive models was employed to investigate the nonlinear relationship between SII and FSD in more detail (Fig. 2). According to our findings, there was a nonlinear relationship between SII and FSD. Upon stratification by smoking status, a U-shaped curve with an inflection point of 2100 (1000 cells/µL) was discerned among the smoker group (refer to Fig. 3 and Table 2), with the same set of variables adjusted for.

### 3.4. Subgroup analyses

We performed stratified subgroup analyses to investigate the relationship between SII and FSD in various population subgroups, considering factors such as age, race, BMI, marital status, hypertension, diabetes, and menstruation. These analyses were carried out using stratified weighted multivariate regression techniques, and interaction effects were assessed (Table 4). While age, BMI, marital status, hypertension, diabetes, and menstruation did not show statistical significance, we found a significant interaction for race (P for interaction < 0.05). In subgroup analyses categorized by ethnic background, our findings indicate that the inverse correlation between SII and FSD is distinctly and significantly affirmative in the Mexican American subgroup [0.9959 (0.9923, 0.9996)], while lacking statistical significance in other racial categories. In summary, our results indicate that the association of SII and FSD exhibits dependence on Mexican Americans, which may be applicable to non-Mexican American populations.

**Table 4:** Association between systemic immune-inflammatory index and female sexual dysfunction in subgroups.

Subgroup		OR (95%CI)	*P* for interaction
Age (yr)			.5017
<50	N = 751	0.9995 (0.9987, 1.0004)	
≥50	N = 189	0.9990 (0.9974, 1.0005)	
Race			<.0001
Mexican American	N = 140	0.9959 (0.9923, 0.9996)	
Other Hispanic	N = 118	0.9977 (0.9930, 1.0025)	
Non-Hispanic White	N = 397	1.0002 (0.9995, 1.0008)	
Non-Hispanic Black	N = 218	0.9989 (0.9975, 1.0002)	
Other Race	N = 66	0.9997 (0.9947, 1.0047)	
BMI (kg/m^2^)			.1366
<25	N = 244	0.9998 (0.9985, 1.0011)	
25–30	N = 210	1.0008 (0.9993, 1.0024)	
>30	N = 485	0.9989 (0.9980, 0.9999)	
Marital status			.1697
Married/living with partner	N = 501	0.9998 (0.9990, 1.0006)	
Widowed/divorced/separated/ Never married	N = 438	0.9989 (0.9978, 0.9999) 0.0308	
Hypertension			.6291
Yes	N = 318	0.9996 (0.9987, 1.0004)	
No	N = 620	0.9992 (0.9982, 1.0003)	
Diabetes			.9414
Yes	N = 99	0.9986 (0.9947, 1.0026)	
No	N = 821	0.9993 (0.9985, 1.0001)	
Borderline	N = 16	0.9789 (0.0000, inf.)	
Menstruation			.4108
Abnormal menstruation	N = 658	0.9992 (0.9983, 1.0001)	
Regular menstruation	N = 281	0.9998 (0.9987, 1.0009)	

The results show that the subgroup analysis was adjusted for all presented covariates except the effect modifier.

95% CI = 95% confidence interval, BMI = body mass index, OR = odds ratio.

## 4. Discussion

Our investigation, involving 1042 participants within a cross-sectional study, revealed that individuals with elevated SII were associated with a reduced likelihood of FSD. Interestingly, we identified a distinct U-shaped correlation between the SII and FSD in the smoking group, characterized by a prominent inflection point occurring at 2100 (1000 cells/µL). Following subgroup analysis and interaction testing, it was revealed that this association exhibited a consistent pattern across diverse demographic settings except for Mexican Americans. Our discovery indicates that elevated SII levels independently pose a risk for reduced FSD within the population experiencing depression.

To our current understanding, this investigation represents the initial exploration of the relationship between SII and FSD. The SII, derived from the enumeration of 3 distinct circulating immune cell types (neutrophils, lymphocytes, and platelets), accurately captures the inflammatory milieu, establishing itself as a discernible biomarker for systemic inflammatory activity.^[27–29]^ Past investigations have scrutinized the association between sexual dysfunction and inflammatory mechanisms. Naoki et al included 71 dialysis patients in a cross-sectional study using the evaluation of erectile dysfunction (ED) conducted using the Sexual Health Inventory for Men (SHIM), which revealed a significant positive association between low-grade systemic inflammation and severe ED.^[30]^ Giugliano et al investigated the relationship between visceral adiposity, chronic low-grade inflammation, and erectile function in a cohort of 80 obese male individuals. This study revealed significant correlations between ED and various inflammatory markers, including but not limited to IL-6, IL-8, IL-18, and CRP, alongside body weight and waist circumference.^[31]^ Zhijie et al conducted a cross-sectional study involving 4116 men aged 20 years and older. Their research indicated a positive association between dietary inflammatory potential, as assessed through the dietary inflammatory index (DII), and the presence of ED in non-institutionalized males residing in the United States.^[32]^ In a study involving 4554 older adults in England, the results suggest that sexually active older adults tend to experience an anti-inflammatory state. This finding is consistent with the negative association between the frequency of sexual activity and inflammatory markers such as CRP and fibrinogen.^[33]^ The current state of research on FSD and its association with inflammation remains inconclusive. However, building upon prior research exploring the connection between inflammation and ED, it becomes apparent that inflammation is significantly linked to sexual dysfunction. Notably, studies have consistently demonstrated inflammation substantial predictive capacity within the domain of sexual dysfunction research. In line with prior research findings on the association between inflammation and the frequency of sexual activity, our examination focused on low-frequency sexual activity, revealing a negative correlation between the SII and FSD. It is essential to highlight, however, that these findings diverge from prior research that established a positive correlation between inflammation and ED. This result suggests that effectively ameliorating or preventing FSD may be achieved through the management of the inflammatory status in patients with depression.

Existing research has consistently demonstrated a significant comorbidity between depression and sexual dysfunction.^[34]^ The literature confirms that the determinant of the presence or absence of sexual dysfunction is primarily depression, as opposed to the burden of physical disease or the severity of complications.^[35]^ A cross-sectional study unveiled a noteworthy link between individuals afflicted by depression with concurrent medical conditions and reduced sexual desire. Furthermore, it revealed a substantial 90% prevalence of sexual dysfunction in females of reproductive age experiencing depression.^[36]^ Besides, the connection between SII and depression is well-established in the scientific literature.^[37]^ The timely recognition of biomarkers for FSD is crucial, serving significant purposes such as prognosis, rehabilitation, and enhancing the overall quality of life among individuals with depression.

Extensive research supports the association between SII and metabolic disorders, as exemplified by Xie et al discovery of a novel correlation between SII and hepatic steatosis and fibrosis in nonalcoholic fatty liver disease (NAFLD), highlighting inflammation pivotal role in NAFLD progression. Independent associations were observed between liver fibrosis, cirrhosis, and higher bone mineral density (BMD).^[38]^ Notably, estrogen deficiency, especially in postmenopausal women, is linked to low BMD, and variations in total testosterone (TT) and free estradiol are associated with a higher NAFLD incidence.^[39]^ This suggests that the shared impact of inflammation and metabolic syndrome may extend to broader physiological implications for sexual function. Furthermore, the role of diet in controlling inflammation is pivotal, with dietary patterns and components known to modulate inflammation in various organs and tissues, and the pivotal role of diet in inflammation control is underscored by the DII. Elevated DII levels, indicative of a diet with higher inflammatory potential, may heighten systemic inflammation, potentially exacerbating SII-related conditions and influencing FSD risk.^[40]^ Additionally, the positive correlation between SII and Abdominal Aortic Calcification (AAC), particularly in older adults, suggests the involvement of cardiovascular factors in the systemic immune response.^[41]^ This integrated perspective, exploring the role of both mediated effects on the association between SII and FSD that dietary choices captured by DII and cardiovascular health indicated by AAC, offers valuable insights into the nuanced relationship between inflammation, cardiovascular factors, and FSD, enhancing our understanding of how lifestyle and cardiovascular health intersect to shape the association between SII and sexual function.

Pain is a prominent clinical manifestation of inflammation.^[42]^ Therefore, women experiencing significant pain during intercourse show elevated inflammation markers, both locally in the genital and vaginal tissues and systemically.^[43,44]^ Elevated inflammation is linked to a range of behavioral and psychological consequences, including fatigue and diminished sensory stimulation.^[16]^ This could imply that women experiencing increased inflammation might develop a higher pain threshold during intercourse, which may, in turn, lead to more frequent sexual activity. On the other hand, inflammatory mechanisms may induce heightened blood perfusion to sexual organs, consequently augmenting comfort levels during sexual intercourse. The mechanism at play bears resemblance to the modus operandi exhibited by the pharmaceutical agent Viagra (sildenafil). The pharmaceutical agent Viagra functions through inhibiting phosphodiesterase and protein phosphatase activities and simultaneous stimulation of β-receptors, which may incite an inflammatory response, subsequently elevating libido.^[45]^ Inflammation can exert a profound influence on the central nervous system, modulating essential brain regions responsible for orchestrating sexual arousal. These regions encompass the mesolimbic reward circuitry, the cingulate cortex, and the thalamic nuclei.,^[46]^ which could heighten the perception of sexual stimulation by sexual organs, thereby elevating libido.

The ethnic variation in the relationship between elevated SII levels and reduced FSD observed in the study may stem from a complex interplay of genetic, cultural, and lifestyle factors among different ethnic groups.^[47]^ Genetic differences may contribute to varying inflammatory responses, while cultural and lifestyle factors could interact differently with inflammation across diverse demographics. The exclusion of Mexican Americans from the consistent pattern suggests unique factors within this group influencing the SII-FSD relationship. Further research, including genetic and cultural investigations, is essential for a more concise understanding of how inflammation impacts sexual function across ethnicities.

Our investigation possesses various notable advantages. Primarily, as we employed a nationally representative sample, our research captures the characteristics of a demographically heterogeneous cohort of adult individuals within the United States, encompassing various ethnicities and ages. Secondly, we meticulously controlled for confounding covariates, thus enhancing the robustness and generalizability of our findings to a broader spectrum of individuals. Nonetheless, it imperative to acknowledge the limitations inherent in our study. Initially, due to the cross-sectional nature of our study design, establishing a causal relationship between SII and FSD was unattainable. Furthermore, since the assessment of FSD, as measured by low sexual frequency, relied on personal interviews, the inevitability of recall bias in our study should be acknowledged. In addition, given the stochastic characteristics of missing covariate data, and despite our meticulous adjustment for several pertinent confounders, the complete exclusion of potential confounding factors cannot be guaranteed, warranting consideration for additional variables that may exert influence.

## 5. Conclusion

Higher SII was associated with a decreased likelihood of FSD, as measured by low sexual frequency incidence, in individuals with depression. Our study suggests that addressing and managing SII in patients with depression could potentially lessen or prevent the onset and recurrence of FSD. Nevertheless, a comprehensive understanding of the causal relationship necessitates further investigation through large-scale prospective studies.

## Author contributions

**Conceptualization:** Guangwei Qing, Bo Wei.

**Data curation:** Guangwei Qing, Hao He, Minghao Lai, Xue Li.

**Formal analysis:** Minghao Lai.

**Funding acquisition:** Bo Wei.

**Methodology:** Guangwei Qing, Bo Wei.

**Software:** Guangwei Qing, Hao He, Xue Li.

**Supervision:** Bo Wei.

**Validation:** Minghao Lai.

**Visualization:** Hao He.

**Writing – original draft:** Guangwei Qing, Yan Chen.

**Writing – review & editing:** Bo Wei.
